# Ahead of the Curve: New Insights into Microtubule Dynamics

**DOI:** 10.12688/f1000research.7439.1

**Published:** 2016-03-10

**Authors:** Ryoma Ohi, Marija Zanic

**Affiliations:** 1 Department of Cell and Developmental Biology, Vanderbilt University School of Medicine, Nashville, Tennessee, USA; 2 Department of Chemical and Biomolecular Engineering, Vanderbilt University, Nashville, Tennessee, USA

**Keywords:** Microtubule dynamics, microtubule biochemistry, cytoskeleton

## Abstract

Microtubule dynamics are fundamental for many aspects of cell physiology, but their mechanistic underpinnings remain unclear despite 40 years of intense research. In recent years, the continued union of reconstitution biochemistry, structural biology, and modeling has yielded important discoveries that deepen our understanding of microtubule dynamics. These studies, which we review here, underscore the importance of GTP hydrolysis-induced changes in tubulin structure as microtubules assemble, and highlight the fact that each aspect of microtubule behavior is the output of complex, multi-step processes. Although this body of work moves us closer to appreciating the key features of microtubule biochemistry that drive dynamic instability, the divide between our understanding of microtubules in isolation versus within the cellular milieu remains vast. Bridging this gap will serve as fertile grounds of cytoskeleton-focused research for many years to come.

## Introduction

Microtubules are hollow, cylindrical polymers of αβ-tubulin that are vital for many biological processes, including cell division, polarization, and migration. In cells, microtubules are composed of 13 laterally associated protofilaments, strands of αβ-tubulin subunits lined up in a head-to-tail fashion. Key to their functional versatility is that microtubules are capable of assembling and disassembling over many cycles, as first revealed by Shinya Inoue’s seminal polarization microscopy studies of mitosis ^1^. The dynamic nature of microtubules is intrinsic to tubulin, as microtubules formed *in vitro* with tubulin alone coexist as growing and shortening polymers that switch between these states ^2^. This non-equilibrium behavior, termed dynamic instability, is in turn dependent on the ability of β-tubulin to bind and hydrolyze GTP: an αβ-tubulin heterodimer that contains GTP-β-tubulin can add on to the growing end of a microtubule, but polymerization induces GTP hydrolysis. GDP-β-tubulin is restricted to the lattice, as ends rich in GDP-β-tubulin are unstable and prone to depolymerization. These observations laid the foundation for the “GTP cap” model, which postulates that microtubules can grow only when non-hydrolyzed GTP-β-tubulin subunits crown the end of a microtubule ^3, 4^. Although GTP hydrolysis plays an indisputable role in fueling dynamic instability, structural aspects of microtubule subunit interactions—both longitudinally and laterally—ultimately lie at the heart of microtubule dynamics.

Whereas the general principles of dynamic instability are well established, individual aspects of a microtubule’s life—nucleation, growth, and the growth-to-shortening transition (catastrophe)—are complex and represent the output of poorly understood multi-step processes. In this commentary, we review recent progress in the field, focusing on nucleation and microtubule assembly, where significant advances have been made. This progress reflects our improved understanding of key microtubule-associated proteins (MAPs), development of *in vitro* assays that probe novel aspects of microtubule assembly and disassembly, and technological breakthroughs that have increased the resolving power of cryo-electron microscopy (cryo-EM)-based structural approaches.

## Nucleation

For a microtubule to form, it must first be *nucleated*. What does this mean in the context of microtubules? A nucleus is a multimer that forms through sequential subunit addition and allows growth to be thermodynamically favorable ^5^. Nuclei can assemble in the absence of cellular factors and have been observed by EM to be two-dimensional sheets ^6, 7^ or short oligomers ^8^, which presumably grow until tube closure is possible. Such spontaneous nucleation is a slow and energetically unfavorable process, involving a considerable lag phase. Importantly, cells bypass the kinetic barrier to nucleation by using factors that accelerate microtubule formation.

The best-understood microtubule nucleation factor is a protein complex that contains γ-tubulin, a protein closely related to α- and β-tubulin. The γ-tubulin ring complex (γ-TuRC) specifically templates 13-protofilament microtubules ^9^ and participates in microtubule nucleation at microtubule-organizing centers such as the centrosome, as well as in the chromosome-mediated ^10– 12^, Golgi-mediated ^13, 14^, and microtubule-dependent ^15– 17^ microtubule nucleation pathways. The γ-TuRC fulfills the expected function of a microtubule nucleating factor; that is, microtubules form more rapidly in its presence ^18^, and it is easy to imagine how it does so: the γ-TuRC mimics an early assembly intermediate of αβ-tubulin ^19^. However, studies of the chromosome-mediated microtubule nucleation pathway have demonstrated that two additional MAPs—TPX2 ^20^ and XMAP215 ^21^—also play key roles in facilitating microtubule nucleation ^22, 23^. In fact, these proteins can nucleate microtubules independently of the γ-TuRC ^23, 24^ when added to concentrations of tubulin at which polymer does not form. As MAPs, the γ-TuRC, TPX2, and XMAP215 have distinct biochemical properties. XMAP215, through the concerted activities of an array of tubulin-binding TOG domains, processively catalyzes microtubule assembly ^25^, increasing microtubule growth rates up to 10-fold ^26^. This activity depends on the ability of XMAP215 to bind tubulin subunits while retaining a grip on the microtubule plus end ^27^. TPX2, on the other hand, has not been reported to bind tubulin subunits and therefore seems to be a conventional MAP that stabilizes and crosslinks microtubules ^24^. On a molecular level, these findings suggest that multiple non-redundant activities can be integrated during microtubule nucleation, providing support that nucleation is a multi-step process. Interestingly, TPX2 and XMAP215 interact with proteins that co-locate their activities with the γ-TuRC at the centrosome. TPX2 binds RHAMM ^28^, which is present in a microtubule nucleation complex that contains γ-TuRC, and NEDD1, a centrosome-targeting factor ^29^. XMAP215 family proteins are targeted to the centrosome through direct interaction with a group of coiled-coil proteins called TACCs ^30^. Therefore, it is likely that TPX2 and XMAP215 family proteins synergize with γ-TuRC in the context of the cell, but how they do so remains unclear.

At a molecular level, recent studies have shed light on how TPX2 and XMAP215 promote nucleation ^31, 32^. Previous *in vitro* work showed that TPX2 induces the formation of disordered tubulin aggregates, which were speculated to be small oligomers capable of elongation ^24^. This finding has recently been recapitulated by the Surrey laboratory by using total internal reflection microscopy-based assays; TPX2 induces the formation of granular tubulin foci (“stubs”), which elongate in solution ^31^. In addition, Roostalu *et al.* show that chTOG, the human ortholog of XMAP215, only mildly promotes microtubule nucleation ^31^. Interestingly, this result differs from conclusions reached through previous work, where XMAP215 alone was sufficient for robust microtubule formation ^23^. However, it is worth noting that the ability of XMAP215 to nucleate microtubules in the previous study was dependent on its conjugation to beads, an experimental setup that locally concentrates the protein. Strikingly, Roostalu *et al.* find that a combination of chTOG and TPX2 produces more microtubule polymer, leading to the idea that chTOG and TPX2 perform distinct functions during the nucleation process. The authors speculate that TPX2 promotes nucleation by stabilizing early oligomeric intermediates, whereas chTOG acts by accelerating subunit addition to nuclei ^31^. chTOG activity may be crucial for oligomers to form a sheet large enough to fold into a tube. In this view, the γ-TuRC may simply act to ensure that microtubules are built using 13 protofilaments, as microtubules nucleated by TPX2 and chTOG are likely of mixed protofilament composition.

Whereas nucleation *de novo* can easily be imagined to be a multi-step process, microtubule assembly on pre-existing templates such as axonemes is also complex, as initially demonstrated by Walker *et al.* ^33^. This problem was revisited in a recent article from the Brouhard laboratory ^32^. Wieczorek *et al.* found that a lag phase always precedes microtubule assembly, regardless of whether the nucleating source is a centrosome, axoneme, or a pre-formed microtubule end ^32^. This lag phase is attenuated by TPX2 and XMAP215, or extended by catastrophe factors such as MCAK and EB1. Although the Roostalu *et al.* and Wieczorek *et al.* studies demonstrate a role for TPX2 and XMAP215 in microtubule nucleation, it is important to note that the two proteins may act differently during templated nucleation versus ** microtubule formation *de novo*. Both reports show that TPX2 is a strong anti-catastrophe factor that slows depolymerization ^31, 32^. Therefore, it is possible that TPX2 acts as a traditional MAP during templated nucleation, simply increasing a filament’s probability to extend. Mechanism aside, the picture we are left with is that the birth of a microtubule is complex, requiring the formation of a plus-end structure that is compatible with subunit addition.

## Microtubule assembly and tubulin structure

Once formed from a nucleus, the growing microtubule will continue to *elongate*, sometimes for minutes at a time. Our understanding of the structure of the elongating microtubule end is shaped by early cryo-EM studies ^34, 35^. These images show that growing microtubule ends display “sheets” of interconnected protofilaments, which are thought to dynamically close into a tube as the microtubule grows. After more than 20 years, direct visualization of the structure of growing microtubule ends in real time remains an open challenge.

The highest-resolution studies of microtubule growth dynamics to date employed optical trapping methods to observe changes in microtubule length with up to 3.5 nm resolution ^36, 37^. In these experiments, microtubules were grown against barriers, such that the observed length fluctuations represent the length changes of the longest individual protofilament or group of protofilaments, and do not uncover the structure of the end. Nevertheless, these studies showed that microtubule growth is irregular with frequent shortening excursions that can retract the longest protofilaments more than 40 nm (corresponding to the length of five tubulin dimer subunits) while a microtubule remains in the overall growth phase.

Large fluctuations in polymer length observed during microtubule growth can be understood as a consequence of a very unproductive growth process ^38^. Indeed, further studies by Gardner *et al.* ^39^ found that the vast majority of tubulin subunits that associate with the growing microtubule end rapidly dissociate. The underlying cause of this high tubulin off-rate is unclear; efficient subunit incorporation into the microtubule lattice might require an additional step (for example, a structural alteration that would promote formation of stabilizing lateral bonds). The exact structure of tubulin subunits when bound to different nucleotides, both in solution as well as within the microtubule polymer, has been somewhat controversial. Whereas earlier studies proposed that GTP-tubulin is straight ^40– 42^, allowing it to readily incorporate into the microtubule lattice, more recent studies suggest that GTP-tubulin dimers are curved, similar to their hydrolyzed, GDP-bound counterparts ^43– 45^. Additionally, detailed structural changes that accompany GTP hydrolysis once a subunit is incorporated in the microtubule polymer have, until recently, been unknown.

A recent study by Alushin *et al.* ^46^ used high-resolution cryo-EM, combined with computational modeling, to investigate the effect of GTP-hydrolysis on the structure of tubulin dimers within the microtubule lattice. With a 5Å resolution, the authors report that GDP-bound tubulin dimers undergo longitudinal compaction close to the exchangeable nucleotide site with tubulin dimers within microtubules grown with a slowly hydrolyzable GTP-analog GMPCPP. In contrast to a previous lower-resolution EM study, performed with a different nucleotide analog (GTPyS) ^47^, Alushin *et al.* found no evidence for changes in lateral interactions between the tubulin dimers. Rather, the authors hypothesize that the observed structural rearrangements in the intermediate domain and the H7 helix of α-tubulin increase lattice strain, which ultimately results in microtubule lattice destabilization. A new study by Geyer *et al.* ^48^ supports the idea that structural changes associated with GTP-hydrolysis underlie microtubule instability. Here, the authors studied the polymerization dynamics of purified yeast tubulin with a mutation in helix H7 of β-tubulin (T238A), which is expected to block H7 movement upon nucleotide hydrolysis. Although the GTPase activity of these microtubules was unaffected, the mutation indeed appeared to prevent structural changes that accompany hydrolysis. Interestingly, microtubules assembled from T238A tubulin are hyperstable, suggesting that allosteric effects of GTP hydrolysis, rather than hydrolysis itself, drive microtubule instability. Future studies with additional tubulin mutants are likely to provide a more detailed link between tubulin structure and microtubule dynamics.

## Microtubule-associated proteins recognize and modulate microtubule structure

Structural features at the microtubule plus end also govern the action of MAPs. XMAP215, for example, is thought to promote microtubule assembly by tethering a weakly bound tubulin dimer to the microtubule end until it becomes stably incorporated into the microtubule lattice ^25^. In the absence of soluble tubulin, XMAP215 is thought to convert a tightly bound subunit at the microtubule end into one that is only loosely associated. This can explain why XMAP215 can promote microtubule depolymerization ^49^ in addition to assembly. Indeed, recent structural studies with TOG domains of Stu2, the yeast homolog of XMAP215, report that TOG domains preferably bind the curved GTP-like conformation of tubulin ^50^. The authors propose that the TOG domain dissociates from the tubulin dimer once it straightens, a structural transition that presumably accompanies its stable incorporation into the microtubule lattice. This “hand-off” mechanism in turn allows the TOG domain to move forward and processively add the next tubulin dimer. Thus, XMAP215 is thought to bind a specific curved conformation of tubulin dimers expected to be found only at the very end of the growing microtubule.

EB proteins comprise another major family of proteins known for their ability to bind growing microtubule ends. *In vitro* studies with nucleotide analogs established that EBs recognize the nucleotide state of tubulin dimers in the microtubule, preferentially binding to GTP-like tubulin over GDP-tubulin ^51, 52^. Interestingly, EBs discriminate between microtubules formed from two GTP mimics—GMPCPP and GTPγS—favoring the latter ^51^. In this context, it is noteworthy that EBs form comets that lag behind the distal microtubule tip occupied by XMAP215, both *in vitro* and in cells ^53, 54^. It is thus speculated that GTPγS mimics GDP-Pi-tubulin, a post-GTP hydrolysis state wherein phosphate has not yet dissociated.

Given that XMAP215 and EB proteins bind different features of the growing microtubule end, it is interesting that the two proteins synergize *in vitro* to promote fast microtubule growth, with rates matching those previously observed only inside of cells ^55^. This synergy is not realized through direct interaction between XMAP215 and EB1. Rather, it is due to an allosteric interaction involving the microtubule end structure. The authors hypothesized that EBs induce structural changes, such as protofilament straightening, that could in turn promote lateral protofilament interactions and sheet closure. Such EB-induced structural changes at the plus end could increase the polymerase activity of XMAP215 by accelerating subunit “hand-off” ^50^.

Previous studies have reported that EB family proteins can affect the structure of the microtubule lattice ^56^ as well as modulate the number of microtubule protofilaments ^47, 57^. Binding of EBs at the interface of four tubulin dimers ^47^ could facilitate such structural effects. The latest evidence that EBs modulate the structure of the tubulin dimers in the microtubule lattice comes from a new study by the Nogales lab ^58^. Here, the authors determined the structures of GMPCPP-, GTPγS-, and GDP-bound microtubules copolymerized with EB3 at an unprecedented resolution of 3.5Å. The authors found all three structures grown with EB3 to be compacted, similar to GDP lattice in the absence of EB3, suggesting that EB binding induces compaction of the microtubule lattice. Unfortunately, the authors were unable to obtain the structure of the GTPγS lattice in the absence of EBs, leaving open the question of whether compaction occurs prior to, or after, phosphate release. In either case, induction of lattice compaction via EBs is consistent with the view that EBs promote GTP hydrolysis in the microtubule lattice.

Allosteric interactions between MAPs are not limited to XMAP215 and EB1 ^59^. A recent study found similar interactions between EB1 and another TOG-domain protein, CLASP ^60^. The authors reported that EBs have a lower binding affinity for microtubules that are grown in the presence of CLASP. The exact features encoded in the microtubule by CLASP that are recognized by EBs remain unknown. Interestingly, the CLASP TOG2 domain exhibits a strongly bent conformation, raising the possibility that CLASP is binding highly curved protofilaments at the microtubule end ^61^. The theme of MAPs recognizing aspects of microtubule curvature is highlighted by other recent studies. Doublecortin, which preferentially binds 13-protofilament microtubules ^62, 63^, enriches on curved microtubule segments ^64^. TPX2 was found to strongly bind curved microtubule ends ^31^. CENP-F associates more strongly with vinblastine-generated tubulin curls compared with straight protofilaments that are found within the microtubule lattice ^65^. Kinesin-5 has been found to associate with curved tubulin protofilaments at growing microtubule ends, where it stimulates microtubule assembly ^66^. Lastly, kinesin-13s, the most potent catastrophe factors known, are well appreciated to recognize and stabilize a bent tubulin protofilament conformation ^67, 68^ observed on depolymerizing microtubule ends ^34, 35^.

## Catastrophe

Even though the link between GTP hydrolysis, structure, and dynamics might be established, we still do not know what features define the stabilizing cap. The observations that EB end binding is largely lost well before the onset of catastrophe ^47, 53, 55^ might imply that GDP-Pi subunits also confer stability to the growing end, if GTPγS tubulin dimers are indeed to be viewed as a model of GDP-Pi state. In that context, and given that EBs bind very strongly to GTPγS microtubule lattice, and much more weakly to the GDP lattice, it is interesting that the only structural difference observed between these two is a small relative rotation of tubulin dimers along a protofilament, resulting in a different lattice twist ^58^. Whether it is this twist that ultimately leads to the high off-rate of GDP tubulin remains to be understood.

Whatever the stabilizing cap looks like, its loss is a complex process intimately linked to structural features of the microtubule end. Recently, Gardner *et al.* reported that the probability of undergoing catastrophe grows with microtubule age ^69^, a finding that confirms older work performed by Odde *et al.* ^70^. Thus, catastrophe cannot be caused by a single-step mechanism unless catalyzed by protein factors such as kinesin-13 ^69^. The process by which microtubule aging causes catastrophes could involve changes in protofilament numbers and/or structural evolution of the growing microtubule end such as tapering and protofilament curling ^71– 74^. In any case, the fate of the microtubule is likely to be encoded in the structure of its end.

## Closing statements

The work reviewed here has significantly advanced our understanding of microtubule assembly and disassembly, but many questions remain. The complexity of the microtubule cytoskeleton in cells, difficult to capture in reconstitution-based approaches, can and should provide a useful framework for posing further questions. An interesting discrepancy concerning the relationship between microtubule end structure and dynamics, for example, is highlighted by the observation that all protofilaments are curved at the plus ends of microtubules during mitosis ^75^. The implication of this finding is that microtubule growth in cells may be governed by different constraints that permit assembly to occur without a sheet-like intermediate that has been observed *in vitro*. Cell cycle-dependent variations involved in the regulation of microtubule biology are also likely to exist. Recent studies on microtubule nucleation have focused on factors that are principally active during cell division. TPX2, for example, is sequestered in the nucleus during interphase ^76^ and requires Ran-GTP to become active during mitosis ^24^. The factors and mechanisms that regulate microtubule nucleation during interphase remain to be elucidated. Lastly, given that most, if not all, aspects of microtubule dynamics involve multi-step processes, an important challenge will be to understand the emergent properties of the network of MAPs that synergistically modulate the kinetics of microtubule assembly and disassembly in ways relevant for cell physiology.
